# Toxic Shock Syndrome Secondary to Erythroderma: Unraveling the Underlying Triggers

**DOI:** 10.7759/cureus.41023

**Published:** 2023-06-27

**Authors:** Abeer Qasim, Nismat Javed, Abhishrut P Jog, Maryam Soliman, Aam Baqui

**Affiliations:** 1 Internal Medicine, BronxCare Health System, New York, USA; 2 Pulmonary and Critical Care Medicine, BronxCare Health System, New York, USA; 3 Pathology, BronxCare Health System, New York, USA

**Keywords:** streptococcal/staphylococcal toxic shock syndrome, erythroderma, toxic shock syndrome, association between tss and erythroderma, bacterial toxic shock syndrome, tss complications, erythrodermic toxic shock syndrome

## Abstract

Toxic shock syndrome (TSS) is a rare and life-threatening condition characterized by the systemic manifestation of severe infection. It is caused by exotoxin-producing strains of *Staphylococcus aureus* and *Streptococcus pyogenes*. Erythroderma, often described as generalized exfoliative dermatitis, is a rare and severe dermatological condition involving more than 90% of the body surface, identified as an uncommon cause of TSS. Here, we describe a case of a 72-year-old male who presented with signs and symptoms of erythroderma presenting as extensive erythematous scaling and lichenified plaques on multiple body surfaces and later developed TSS.

## Introduction

Toxic shock syndrome (TSS) is a rare medical condition caused by exotoxin-producing strains of *Staphylococcus aureus* and *Streptococcus pyogenes* [1]. The incidence of TSS in the adolescent population is 0.79/100,000 [2]. Two metropolitan and state-wide epidemiologic studies estimated the annual incidence of TSS to be 5.2 and 6.4 per million persons in the USA [3]. TSS presents as a flu-like illness with high-grade fever, vomiting, diarrhea, and muscle weakness and can lead to potentially serious complications, such as adult respiratory distress syndrome, renal failure, disseminated intravascular coagulation, encephalopathy, and cardiomyopathy [4]. Erythroderma is identified as an uncommon cause of TSS, with a challenging clinical presentation. It is a rare dermatological condition involving >90% body surface area. The global incidence is approximately one per 100,000 adults [5]. Prompt recognition and early intervention are crucial in preventing mortality.

## Case presentation

**Figure 1:**
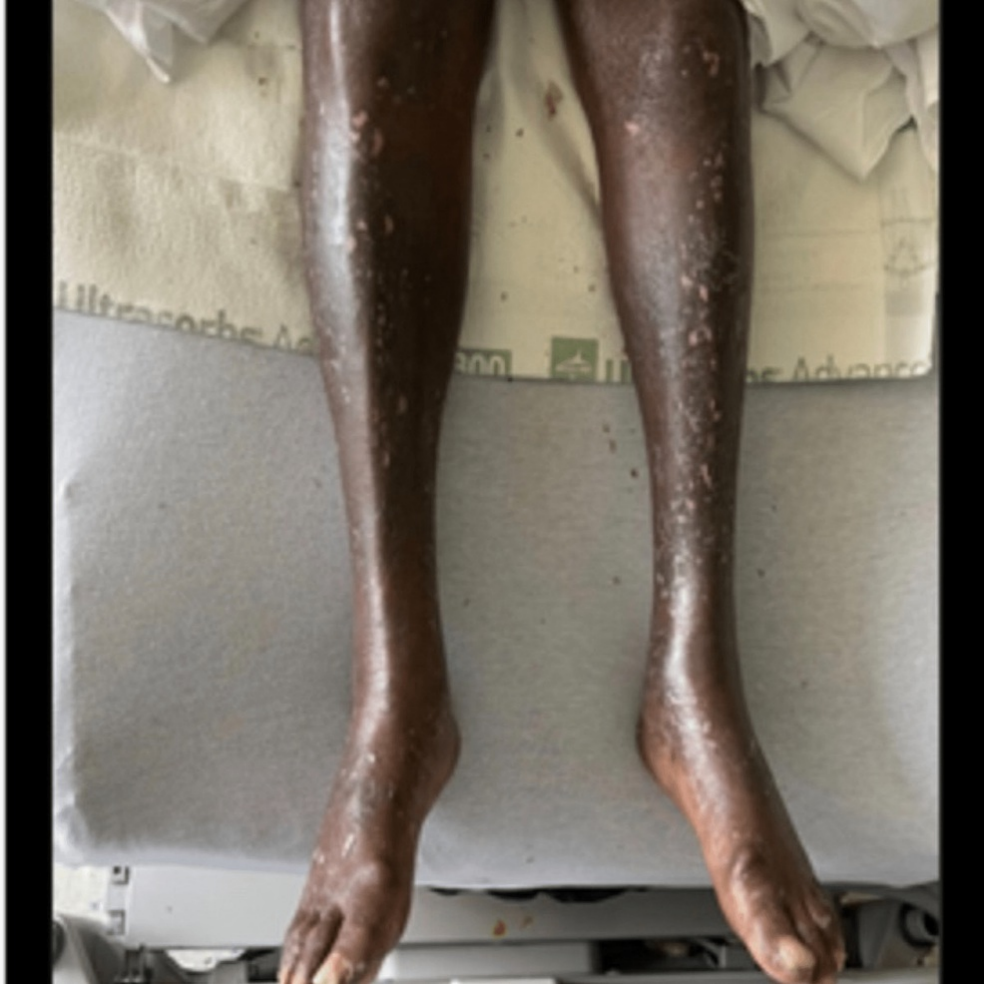
Extensive erythematous scaling involving bilateral lower extremities.

**Figure 2:**
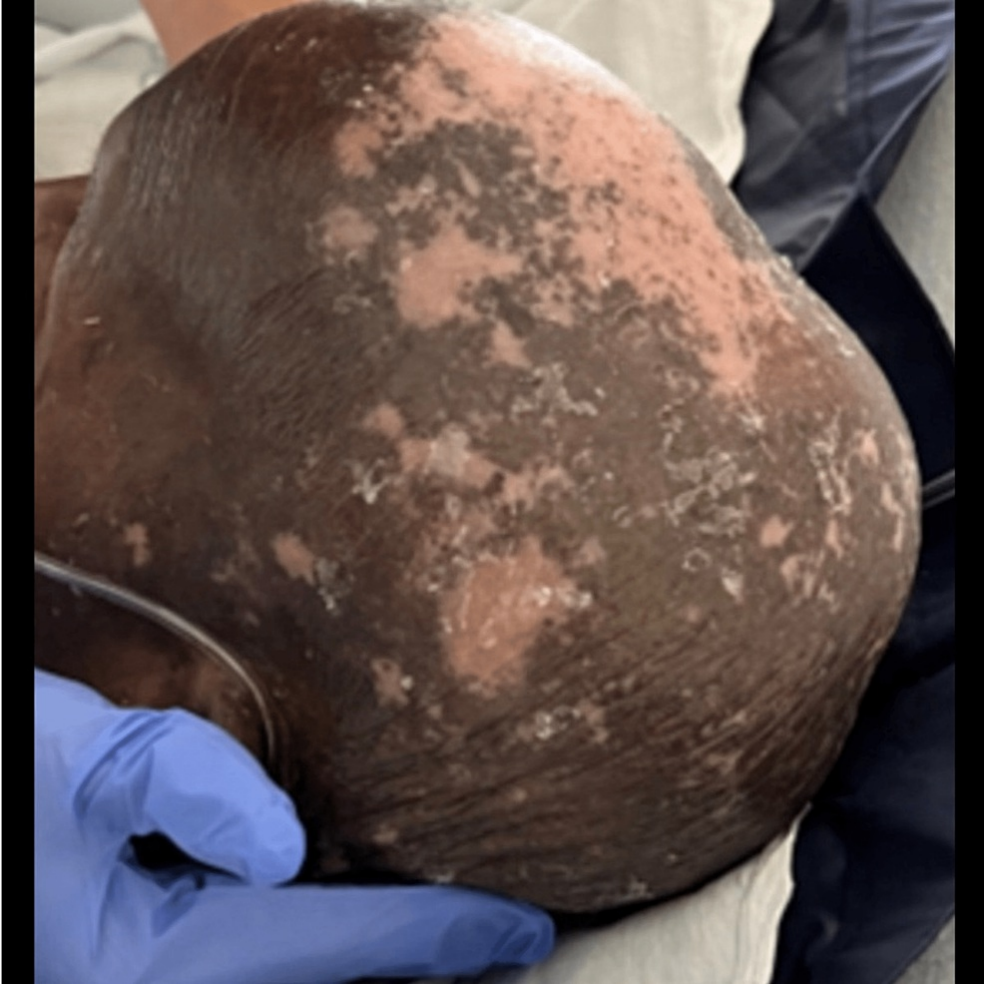
Erythema and scaling of the scalp.

**Figure 3:**
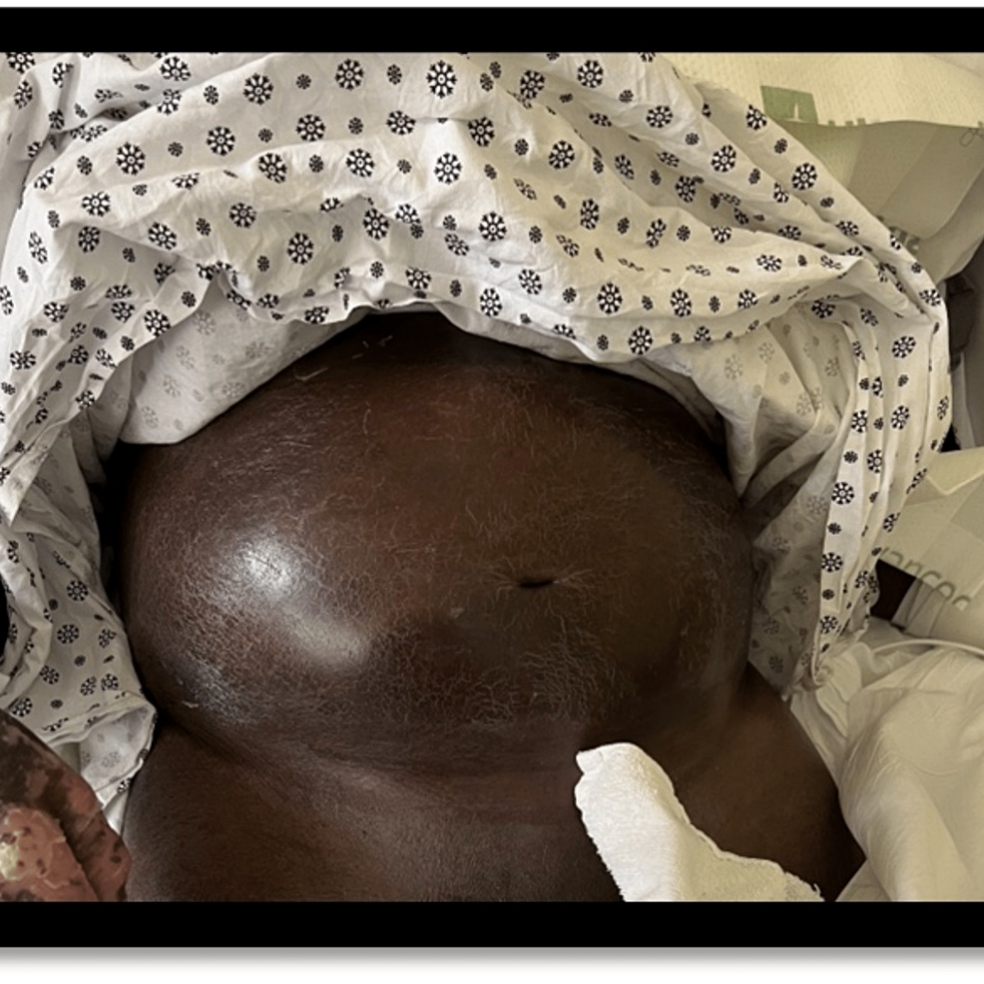
This photo shows erythroderma with diffuse erythema and desquamation of the skin.

**Figure 4:**
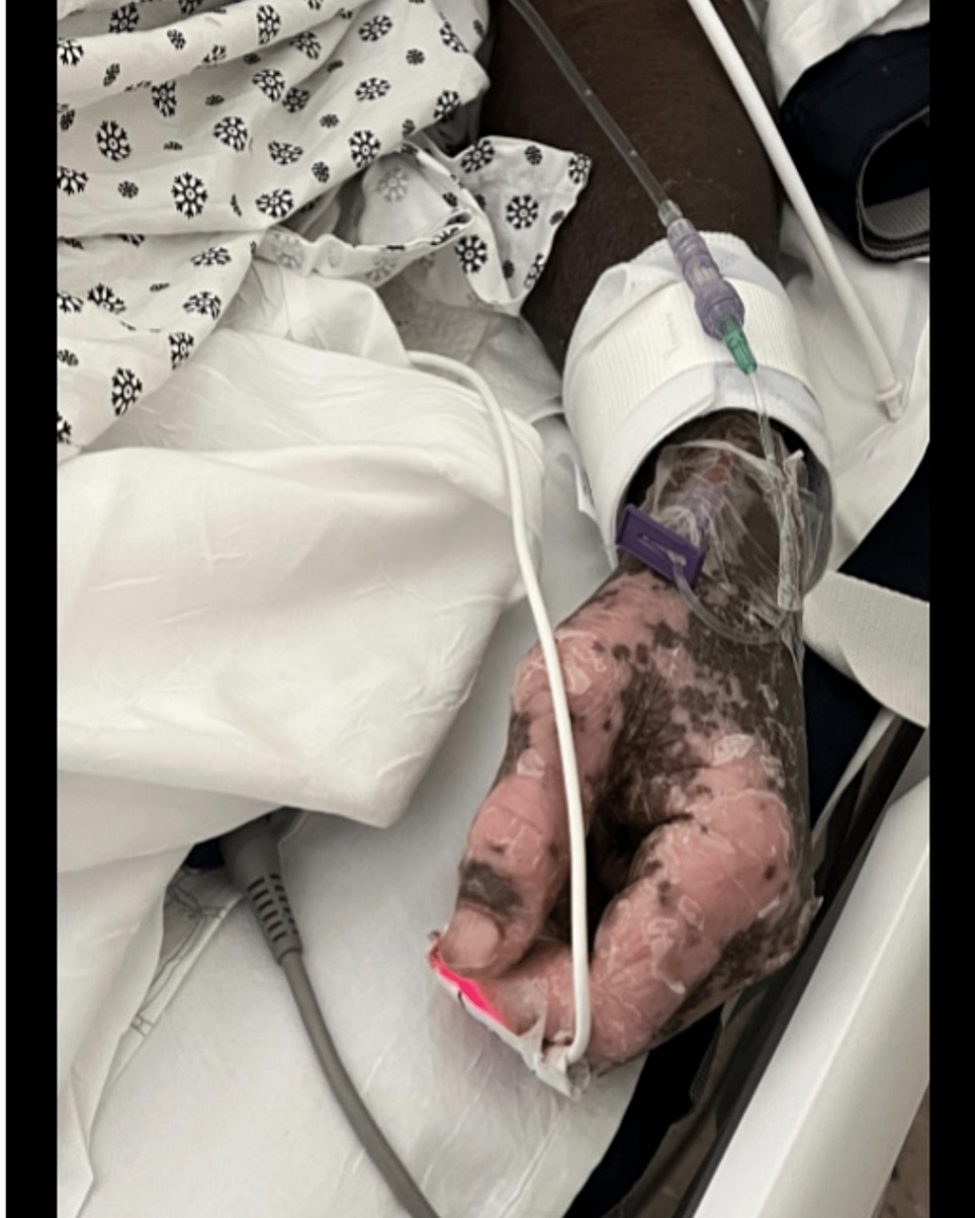
Severe sloughing of the epidermis.

**Figure 5:**
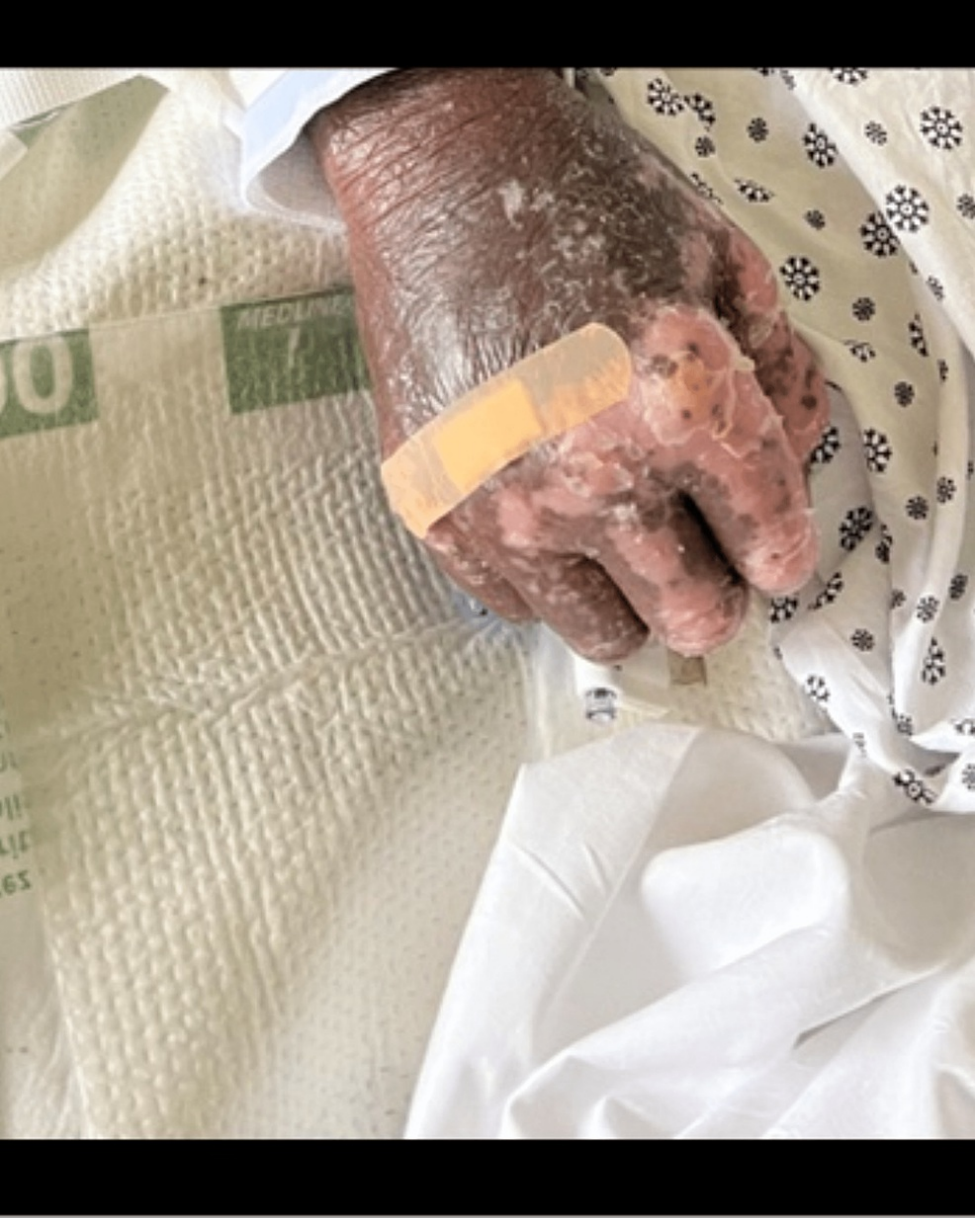
Erythroderma of the hand.

A 72-year-old male with a history of alcohol dependence, eczema, hypertension, and hyperlipidemia presented to the emergency department with a chronic complaint of extensive erythematous scaling and lichenified plaques throughout the body. He stated that he first noticed a pruritic rash on the first two fingers of his right hand 20 years ago, which spread to the other hand, and then progressively involved the entire body, including face, extremities, torso, hands, and feet (Figures 1-5). The rash caused painful skin breakdowns and was aggravated by heat and sunlight. He had tried multiple ointments, which did not alleviate the rash.

On initial arrival, the patient was hemodynamically stable. Physical examination revealed multiple excoriated lesions with sloughing covering a large surface area of the body. Laboratory tests were significant for anemia (11.2 mg/dl), eosinophilia (12.0%), and lactic acidosis (2.1 mmol/L) (Table 1). The rest of the labs were within normal limits. He was admitted to the hospital for intensive treatment and additional investigations involving a skin biopsy.

**Table 1:** Comparison of labs between day one and day five.

Investigations	Value on day 1	Value on day 5	Reference range
WBC count	6.9	12.8 k/ul	4.8-10.8 k/ul
RBC count	3.85	2.39 MIL/ul	4.50-5.90 MIL/ul
Hemoglobin	11.8	7.0g/dl	12.0-16.0 g/dl
Hematocrit	36.2	21.8%	42.0-51.0 %
Mean corpuscular volume	94.1	91.9 fL	80.0-96.0 fL
Platelet	309	21 k/ul	150-400 k/ul
Eosinophil count	0.80	0.00k/ul	0.05-0.25 k/ul
Lactic acid level	2.1	2.5 mmoles/L	.5-1.6 mmoles/L

Five days into the admission, the patient developed altered mental status, hypothermia (94°F), persistent bradycardia (40 beats per minute), and hypotension (88/60 mm of Hg), necessitating admission to the intensive care unit. Repeat labs revealed significant leukocytosis and hypernatremia compared to labs on the day of admission (Table 1).

The patient was resuscitated with fluids and started on vasopressors. Due to concerns for sepsis leading to shock, the patient was started on broad-spectrum antibiotics (vancomycin and piperacillin-tazobactam). The hospital course was complicated by cardiac arrest and acute hypoxemic respiratory failure requiring mechanical ventilation. Blood cultures came positive for methicillin-sensitive *Staphylococcus aureus* (MSSA). Antibiotics were narrowed to nafcillin. The patient responded well to the antibiotics, was tapered off vasopressors, and was successfully extubated. Nafcillin was continued for a duration of four weeks.

**Figure 6:**
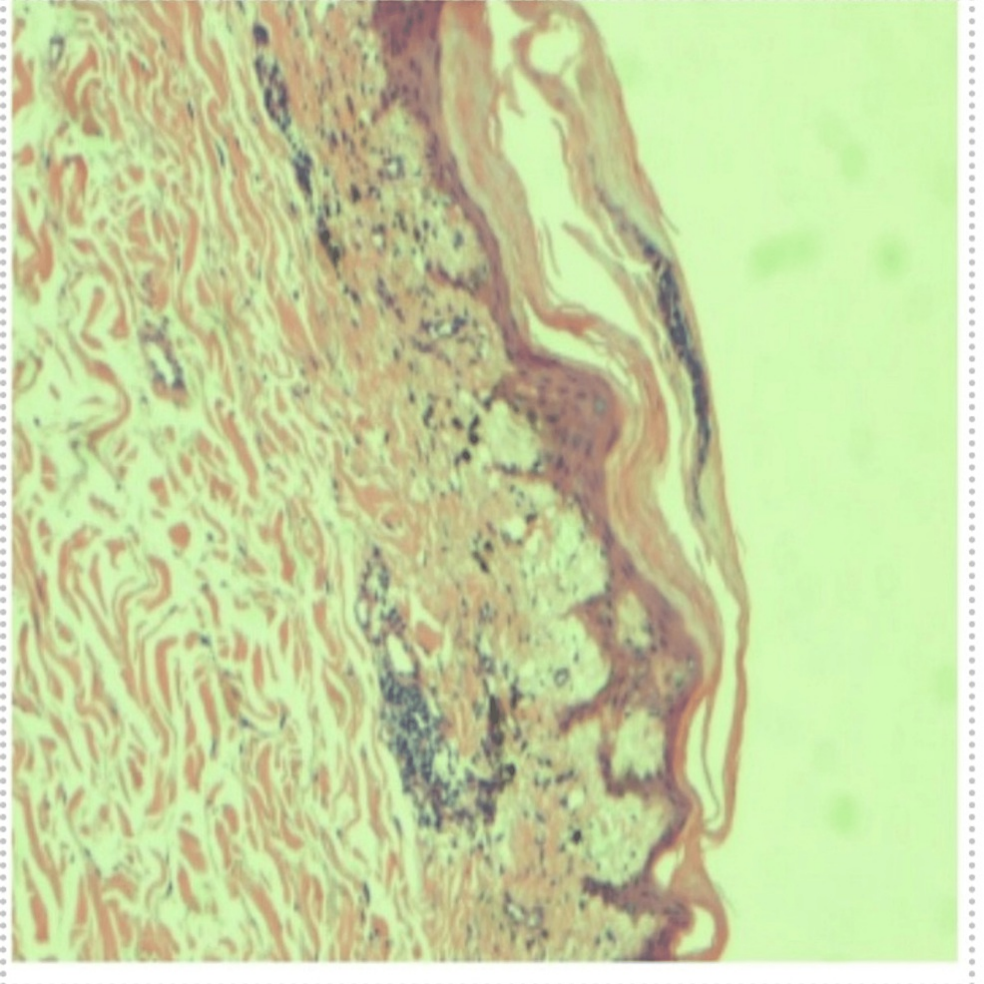
The superficial dermis contains perivascular lymphocytic inflammation and edema. There is hyperkeratosis, parakeratosis, acanthosis, and papillary dermal fibrosis.

**Figure 7:**
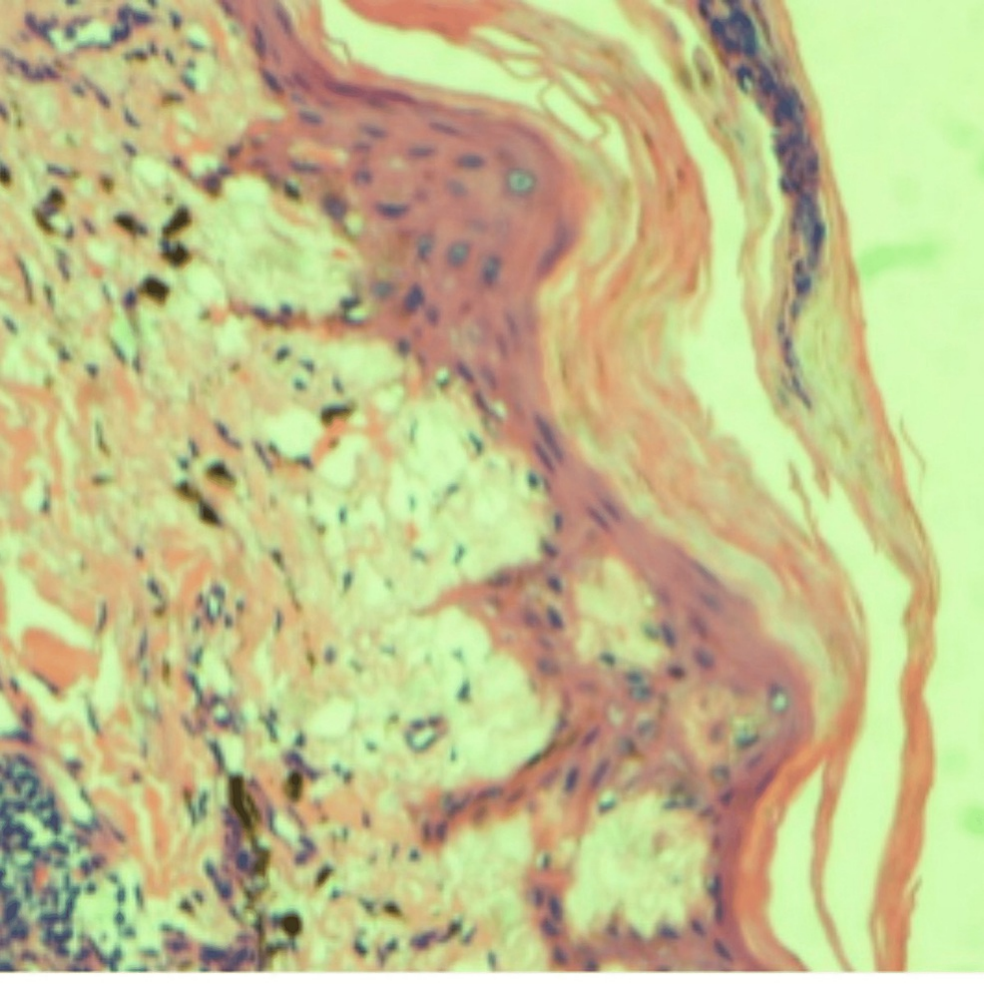
Higher magnification showing post-inflammatory pigmentary alteration.

After stabilization, he underwent a skin biopsy to try to elucidate the underlying cause of the erythroderma. It revealed changes consistent with old interface dermatitides (such as erythema dyschromicum perstans, fixed drug eruption, lichen planus, and collagen vascular disease) (Figures 6, 7).

The presence of eczema in the past was thought to have progressed to erythroderma. Additionally, a review of medications was done to look for medication-induced erythroderma. High-dose atorvastatin was discontinued from the treatment regimen. The patient’s rash and symptoms improved, and he was discharged from the hospital. The patient is following up in the dermatology department as an outpatient, and the rash has improved significantly.

## Discussion

TSS is an entity caused by staphylococcal exotoxin [6]. Organisms known to be causative agents for TSS include *Staphylococcus aureus* and group A *Streptococcus* (*Streptococcus pyogenes*) [7]. A few rare causes have been identified in case reports, including *Streptococcus dysgalactiae* subspecies *equisimilis* and T serotype B3264 streptococcus [8,9]. A few conditions are associated with a higher risk of developing TSS. Table 2 mentions these conditions [10].

**Table 2:** Conditions associated with toxic shock syndrome.

Long-term use of high-absorbency tampons
Soft tissue infections
Post-surgical infections
Burns
Trauma
Retained foreign bodies including nasal packing
Dialysis catheters

In our case, the source of entry for the MSSA was the defect in the skin barrier due to erythroderma. The risk factors for developing erythroderma are listed in Table 3 [11].

**Table 3:** Risk factors associated with erythroderma

Male gender
HIV infection
Herpes simplex virus infection
Drug allergies
Seborrheic dermatitis
Sarcoidosis
Contact dermatitis
Connective tissue diseases
Atopic dermatitis
Autoimmune disorders
Lymphoma and leukemia

The clinical features of TSS include high-grade fever, nausea, vomiting, malaise, headaches, dizziness, hypotension, pharyngitis, peeling of skin in the soles of feet or palms of hands, rash, and multiorgan failure.

In contrast, some clinical features commonly associated with erythroderma include widespread redness, scaling, peeling of skin, itching, swelling, and edema. However, systemic symptoms include fever, chills, fatigue, and malaise. The rash initially presents as gradually expanding red patches with flaking and scaling. Extensive scratching may cause the skin to feel leathery, and there could be involvement around the eyes (periorbital area) [12]. These features can rapidly worsen, as seen in our patient.

Lab investigations in erythroderma show leukocytosis, anemia, eosinophilia, hypoalbuminemia, hyperglobulinemia, and elevated inflammatory markers. Anemia and eosinophilia were observed in our patient. Cultures might also provide additional information about the superimposed infection. *Staphylococcus aureus* has been extensively studied in this regard [13]. The organism serves as an enterotoxin, enhancing superantigenicity and accelerating the rate of progression to TSS [14]. Our patient had tested positive for *Staphylococcus aureus*, which caused TSS.

The management of TSS includes aggressive fluid hydration, identifying necrotizing pathologies early, and removing foreign bodies. Early surgical consultation is warranted in patients needing wound debridement [15]. Antibiotics with a broad spectrum of activity should be given. For MSSA, clindamycin plus flucloxacillin or beta-lactamase-resistant penicillin such as nafcillin is recommended. When dealing with methicillin-resistant *Staphylococcus aureus* (MRSA), vancomycin or linezolid is essential. Clindamycin may also be added [16]. Group A streptococci are usually treated by penicillin [16]. The tentative timeline for the duration of therapy is seven to 14 days.

## Conclusions

The compromised skin barrier in erythroderma can result in skin infection with *Staphylococcus aureus*, which can further cause bacteremia. In some cases, this might lead to TSS. In cases where TSS is suspected, management of aggressive shock and organ dysfunction is critical.
